# Supplementary material for: Publication rates of research projects of an internal funding program of a university medical center in Germany: A retrospective study (2004–2013)
Source: PLoS One. 2020 Nov 30;15(11):e0243092. doi: 10.1371/journal.pone.0243092 (PMC7703943; doi:10.1371/journal.pone.0243092)
Supplement: Attachment — Submitted filename: Response to Reviewers.docx [file pone.0243092.s002.docx]

October 8, 2020

**PONE-D-20-14186**

**“Publication Rates of Research Projects of an Internal Funding Program of a University Medical Center in Germany: A Retrospective Study (2004–2013)”**

Dear Dr. Useche,

Thank you very much for discussing our paper and for the invitation to submit a revised version of our manuscript. We revised the paper according to the reviewers` suggestions and addressed PLOS ONE’s style requirements.

Our replies to the reviewers` comments are detailed on the following pages.

We look forward to hearing from you at your earliest convenience and hope that the revised manuscript is accepted for publication.

Sincerely,

Susanne Deutsch, Silke Reuter, Astrid Rose and René Tolba

For post-publication correspondence please change the corresponding author to René Tolba (Institute for Laboratory Animal Science & Experimental Surgery, RWTH Aachen University, Faculty of Medicine, Pauwelsstraße 30, 52074 Aachen, Germany, email address: rtolba@ukaachen.de). This change has already been made in the revised manuscript.

**Original manuscript: Line 13**

**Revised manuscript with track changes: Line 13**

Review Comments to the Author

**Reviewer #1: 1**. I suggest adding a footnote to Table 1 indicating that Chi square tests were performed with no significance. If tests for trend were also done I would include a footnote referencing that test as well.

**Authors’ response:** We thank the reviewer for the suggestion and added the following footnote to Table 1. No tests for trends were carried out.

^3^ Χ^2^ tests were performed with no significance regarding proposal type, scope of funding, project duration and clinic/institute of the applicant.

**Original manuscript: -**

**Revised manuscript with track changes: Line 180 - 181**

**Reviewer #1: 2.** The sentence starting on line 275 "When questioned..."; I would make it clear that this information comes from previous research (not the current study) and move at least one of the citations: 8,24,25 up to support this statement.

**Authors’ response:** Thank you for this valuable comment. We have revised these sentences.

In previous studies that directly addressed these issues, most researchers cite lack of time and the perception that their research is low priority and not deemed valuable, notably among studies with primarily negative results [24, 25]. Other issues include technical problems and difficulties with co-authors, supervisors and/or peer reviewers [8].

**Original manuscript: Line 275 - 278**

**Revised manuscript with track changes: Line 276 - 280**

**Reviewer #1: 3.** It seems like the first part of reference 8 may have been deleted, please review.

**Authors’ response:** We thank the reviewer for carefully reading the manuscript. However, reference 8 is correctly indicated. It is a reference of Gerben ter Riet, a researcher from the Netherlands.

**Original manuscript: Line 343**

**Revised manuscript with track changes: Line 345**

Again, we would like to thank both the editor and the reviewers for their kind consideration of our manuscript and the opportunity to revise our paper according to their suggestions.

We are very much looking forward to their esteemed analysis of our revision and hope that it may be found suitable for publication in PLOS ONE.
